# Efficacy of therapeutic drug monitoring-based antibiotic regimen in critically ill patients: a systematic review and meta-analysis of randomized controlled trials

**DOI:** 10.1186/s40560-023-00699-8

**Published:** 2023-11-08

**Authors:** Nozomi Takahashi, Yutaka Kondo, Kenji Kubo, Moritoki Egi, Ken-ichi Kano, Yoshiyasu Ohshima, Taka-aki Nakada

**Affiliations:** 1 Centre for Heart Lung Innovation, St. Paul’s Hospital, The University of British Columbia, 1081 Burrard Street, Vancouver, BC V6Z 1Y6 Canada; 2 Department of Emergency and Critical Care Medicine, Chiba University Graduate School of Medicine, Chiba, Japan; 3 Department of Emergency and Critical Care Medicine, Juntendo University Urayasu Hospital, Chiba, Japan; 4 Department of Emergency Medicine and Department of Infectious Diseases, Japanese Red Cross Wakayama Medical Center, Wakayama, Japan; 5 Department of Anesthesia and Intensive Care, Kyoto University Hospital, Kyoto, Japan; 6 Department of Emergency Medicine, Fukui Prefectural Hospital, Fukui, Fukui Japan; 7 Department of Pharmacy, Kobe Tokushukai Hospital, Hyogo, Japan

**Keywords:** Sepsis, Antibiotics, Pharmacokinetics, Pharmacodynamics, TDM

## Abstract

**Background:**

The efficacy of therapeutic drug monitoring (TDM)-based antimicrobial dosing optimization strategies on pharmacokinetics/pharmacodynamics and specific drug properties for critically ill patients is unclear. Here, we conducted a systematic review and meta-analysis of randomized controlled trials to evaluate the effectiveness of TDM-based regimen in these patients.

**Methods:**

Articles from three databases were systematically retrieved to identify relevant randomized control studies. Version two of the Cochrane tool for assessing risk of bias in randomized trials was used to assess the risk of bias in studies included in the analysis, and quality assessment of evidence was graded using the Grading of Recommendations Assessment, Development, and Evaluation approach. Primary outcome was the 28-day mortality and secondary outcome were in-hospital mortality, clinical cure, length of stay in the intensive care unit (ICU) and target attainment at day 1 and 3.

**Results:**

In total, 5 studies involving 1011 patients were included for meta-analysis of the primary outcome, of which no significant difference was observed between TDM-based regimen and control groups (risk ratio [RR] 0.94, 95% confidence interval [CI]: 0.77–1.14; *I*^2^ = 0%). In-hospital mortality (RR 0.96, 95% CI: 0.76–1.20), clinical cure (RR 1.23, 95% CI: 0.91–1.67), length of stay in the ICU (mean difference 0, 95% CI: − 2.18–2.19), and target attainment at day 1 (RR 1.14, 95% CI: 0.88–1.48) and day 3 (RR 1.35, 95% CI: 0.90–2.03) were not significantly different between the two groups, and all evidence for the secondary outcomes had a low or very low level of certainty because the included studies had serious risk of bias, variation of definition for outcomes, and small sample sizes.

**Conclusion:**

TDM-based regimens had no significant efficacy for clinical or pharmacological outcomes. Further studies with other achievable targets and well-defined outcomes are required.

*Trial registration*: Clinical trial registration; PROSPERO (https://www.crd.york.ac.uk/prospero/), registry number: CRD 42022371959. Registered 24 November 2022.

**Supplementary Information:**

The online version contains supplementary material available at 10.1186/s40560-023-00699-8.

## Background

Antibiotic therapy is a cornerstone in the treatment of sepsis and severe infections, and appropriate antibiotic dosing design based on pharmacokinetics/pharmacodynamics (PK/PD) is necessary [1–4]. The Surviving Sepsis Campaign suggested that dosing strategies for antimicrobials should be optimized based on PK/PD and specific drug properties as a best practice statement [5]. However, sepsis can result in an increase in the distribution volume of antibiotics and altered clearance, leading to unpredictable blood levels if treatments are based on normal antibiotic doses [6–11]. In such cases, the usual PK/PD-based dosing regimen may result in inadequate or excessive antibiotic concentrations, leading to poor clinical outcomes or organ damage such as kidney injury [12–14].

Therapeutic drug monitoring (TDM), which refers the management and adjustment of the patient's drug dosing based on the measured drug concentration in the blood, has been suggested as a method of drug exposure optimization [15, 16]. Previous studies have advocated for the importance of TDM to maximize antimicrobial effectiveness and decrease adverse event. However, only few studies have clearly verified the efficacy of TDM-based antibiotic regimen in critically ill patients, regardless of the antimicrobial agent type [17].

Recent systematic review and meta-analysis evaluating TDM for beta-lactam antibiotics included five randomized control trials (RCTs), but two large RCTs on TDM have since been published, increasing the need for reevaluation [18]. Hence, we conducted a systematic review and meta-analysis of RCTs to evaluate the efficacy of TDM-based regimens in critically ill patients, focusing on both clinical and pharmacological outcomes, which includes target attainment. Studies were not restricted by type of antimicrobial agent as the primary aim was to validate the implementation of TDM-based regimens.

## Materials and methods

### Study design and definition

This systematic review and meta-analysis followed the Preferred Reporting Items for Systematic Reviews and Meta-Analyses statement [19], and the protocol for this systematic review was registered in the PROSPERO database on 24 November 2022 (CRD 42022371959). In the protocol, critically ill patients were defined as those meeting any of the following: (1) patients admitted to an intensive care unit (ICU) and requiring artificial support including invasive mechanical ventilation; (2) patients who were considered critically ill based on the definition in each study and had suspected or proven infection, or (3) patients diagnosed with sepsis. Sepsis was defined as Sepsis-3 or severe sepsis with Sepsis-1 or 2, which indicates organ dysfunction due to infection [20]. TDM was defined as the measurement of antimicrobial agent concentration in blood, serum, or plasma at a specific time point, and the dosing adjustment was based on the concentration.

### Research question and inclusion criteria

Our research question was whether TDM should be used to adjust antimicrobial doses in patients with sepsis or treated in the ICU. Accordingly, RCTs comparing TDM to standard procedures in patients aged ≥ 18 years with either sepsis or who were critical ill were included. TDM studies that did not have a control were excluded. Case series, case reports, editorials, letters to editor, and conference abstracts were also excluded from this review.

### Literature search

Two authors (N.T. and Y.O.) independently and systematically searched the MEDLINE (via PubMed), Cochrane CENTRAL databases, and Igaku-Chuo-Zasshi for peer-reviewed articles published as well as the ClinicalTrials.gov and World Health Organization International Clinical Trial Registry Platform for relevant completed trials between database inception and November 2022 (search date: 17 November 2022).

Search formulas were created based on “Sepsis”, “Systemic Inflammatory Response Syndrome”, “Multiple Organ Failure”, “Critically ill”. “Therapeutic Drug Monitoring”, and “Randomized controlled trial” with Medical Subject Heading terms and text words along with a query starting with “NOT” to exclude ineligible studies. The terms were then arranged and entered in a form suitable for each database (Additional file 1: Table S1).

### Screening and data extraction

After extraction of the study list and exclusion of duplicate studies, two authors (N.T. and Y.O.) independently screened the titles and abstracts of all articles to identify eligible studies by using a study by Ryyan [21] as the first screening. If there was conflict between the authors and the disagreement persisted, a third author (Y.K.) was consulted to resolve the conflict through discussion. The second screening was reviewing full texts of the articles included in the final selection, which was independently performed by two authors (N.T. and Y.O.). Subsequently, two authors (N.T. and Y.O.) independently extracted all relevant information from the included studies, including study settings and period, design, year, study population, sample size, patient demographic, type of antibiotics regimen, intervention algorithm and strategy, minimum inhibitory concentration (MIC), target attainment, primary outcomes, and included mortality type.

### Risk of bias assessment using the grading of recommendations assessment, development, and evaluation (GRADE) approach

Two authors (N.T. and Y.O.) independently assessed the risk of bias for each included study using the Cochrane tool for assessing risk of bias in randomized trials, version two (RoB 2) [22]. This tool assesses bias arising from the randomization process due to deviations from intended interventions and missing outcome data in outcome measurement and selection of reported results by classifying each domain with low, intermediate, or high risk of bias. Any conflicts were resolved through a discussion or independent evaluation by a third author (Y.K.).

Quality assessment of evidence was graded using the GRADE system that provides a systematic approach for each outcome across studies [23]. The GRADE system has four levels for the evidence: very low, low, moderate, and high. GRADE scores were reduced by risk of bias, imprecision, inconsistency, indirectness, and publication bias. Publication bias was visually assessed by evaluating the funnel plots [24].

### Outcomes

The primary outcome of interest was 28-day mortality. Secondary outcomes were target attainment defined in each study at day 1 and 3, clinical cure (resolution of infection signs and symptoms, no clinical failure, or as based on definition in each study), hospital mortality, and length of stay in the ICU. If TDM was simultaneously performed for multiple antibiotics, outcomes including target attainment were analyzed separately for each antibiotic. We also performed the analysis by the subgroup for the class of antibiotics when there was more than one study.

### Statistical analysis

Extracted data were collected and statistically analyzed using RevMan ver 5.4.1 (The Nordic Cochrane Centre, The Cochrane Collaboration, Copenhagen, Denmark). Risk ratios (RRs) and corresponding 95% confidence intervals (CIs) were determined using a random-effects model with weights calculated using the Mantel–Haenszel method [25]. For continuous values, mean difference and 95% CIs were calculated using inverse variance-weighted method. Heterogeneity of the included studies was assessed using the estimated Cochrane Chi-square test, Tau^2^, and *I*^2^ statistic, in which *I*^2^ > 70% indicated severe heterogeneity. To calculate point estimates, when the mean and median were different, the mean was considered the same as the median, and the standard deviation (SD) was integrated by dividing the interquartile (IQR) by 1.35 (https://handbook-5-1.cochrane.org/chapter_7/7_7_3_5_mediansand_interquartile_ranges.htm).

A two-tailed *P* < 0.05 for hypothesis and < 0.1 for heterogeneity testing was considered statistically significant.

## Results

**Fig. 1:**
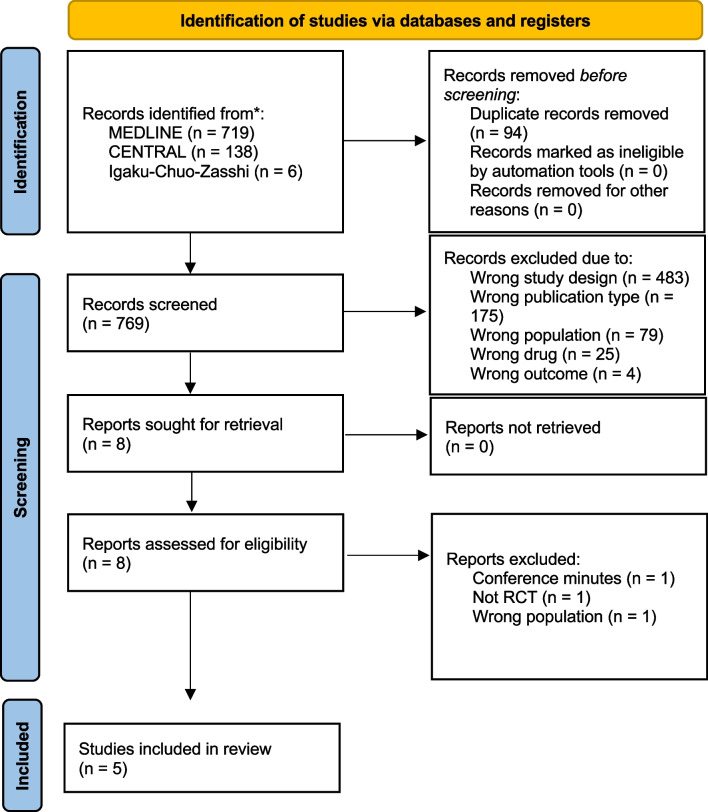
Flow diagram of the study selection

We identified 863 records from searching the three electronic databases. Of these, 769 publications were screened, and 8 studies were retrieved for full text review. Finally, 5 RCTs were included in our meta-analysis (participants, *n* = 1011; TDM group, *n* = 510; standard dosing group, *n* = 501) [26–30] (Fig. 1, Table 1). Four of the studies were conducted in Europe, three were published in 2022; four included beta-lactam antibiotics, two included quinolones, one included aminoglycoside, and one included vancomycin. There were no conflicts of interest reported in any of the studies. The PK/PD targets in the intervention groups differed across the studies. For beta-lactam, the target concentration was set at > 4 × MIC in three studies [27–29] and > 1 × MIC in one study [30], and the dosing was designed to achieve 100% *f*T > 4 × MIC or 100% *f*T > MIC, respectively. In these protocol, 100% *f*T > 4 × MIC means that the free drug concentration exceeds four times the MIC at 100% of the dosing interval. For aminoglycoside, peak and trough concentrations were used as the target, whereas area under the curve/minimum inhibitory concentration (AUC/MIC) was used for vancomycin and quinolones. TDM was not performed simultaneously for multiple antibiotics in any of the identified studies or in those found on the ClinicalTrials.gov and World Health Organization International Clinical Trial Registry Platform.

**Table 1:** Characteristics of included studies in the meta-analysis

First author, year, country	Study settings, period	Study population (TDM/control)	APACHE II score (TDM/Control)^1)^	Major focus of infection	Antibiotics type	Target for intervention	Primary outcome/mortality follow-up
Bartal, 2003, Israel	Single ICU, 1999–2000	*N* = 81; nonsurgical sepsis patients with suspected or documented Gram-negative infection (43/38)	20/18 (amikacin) and 10/11 (gentamicin)	Urinary tract (24.7%)	Aminoglycoside	Target peak; 20 μg/mL (gentamicin) or 60 μg/mL (amikacin), target trough; < 1 μg/mL	Nephrotoxicity/28-day mortality
Waele, 2014, Belgium	Single ICU, 2011–2012	*N* = 41; ICU patients who needed for antibiotic treatment with piperacillin/tazobactam and/or meropenem and the presence of an arterial catheter (21/20)	19/17	Pneumonia (78%), peritonitis (12%)	Piperacillin/tazobactam and/or meropenem extended infusion	Target trough; > 64 mg/L (piperacillin/tazobactam) or > 8 mg/L (meropenem) (> 4 × MIC) and 100%*f*T > 4 × MIC^2)^	Target attainment defined as 100%*f*T > MIC and 100%fT > 4MIC within the first 24 h and 72 h of treatment/hospital and 28-day mortality
Ewoldt, 2022, Netherland	8 ICUs, 2018–2021	*N* = 388; ICU patients who expected to receive the target antibiotics for at least 2 days (189/199)	71/70 (APACHE IV)	Pulmonary (65.2%), intra-abdominal infection (16.0%)	Beta-lactam and ciprofloxacin	Target; 100%*f*T > MIC (beta-lactam) or AUC_0–24 h_ / MIC > 125 (ciprofloxacin)^3)^, above target trough; > 10 × MIC (beta-lactam) or AUC_0–24 h_/MIC > 500 (ciprofloxacin)	ICU length of stay/28-day mortality, ICU mortality, hospital mortality, 6-month mortality
Hagel, 2022, Germany	13 ICUs, 2017–2019	*N* = 249; sepsis patients (125/124)	23/22	Pneumonia (62.2%), intra-abdominal infection (19.7%), urinary tract (12.9%)	Piperacillin/tazobactam as continuous infusion	Target concentration: > 4 × MIC	Sepsis-related organ dysfunction, discharge from the ICU or death/ 28-day mortality
Roggeveen, 2022, Netherland	2 ICUs, 2018–2020	*N* = 252; intensive care patients who received antibiotics for a suspected or confirmed infection and had a suspected or measured serum lactate greater than 2 mmol/L or a requirement for vasopressor support (132/120)	No severity score	No information for focus of infection	Vancomycin, ciprofloxacin, meropenem, and ceftriaxone	AUC_0-24_/MIC > 400 (vancomycin), AUC_0-24_/MIC > 125 (ciprofloxacin), 100%*f*T > 4 × MIC (beta-lactam)	PK target attainment during the first 24 h following randomization/ ICU, hospital, 28-day and 6 months mortality

^1^APACHE, Acute Physiology and Chronic Health Evaluation; values are described by mean or median

^2^*f*T > MIC, time during a dosing interval that the free drug concentration of antibiotics exceeded the MIC

^3^AUC_0-24 h_/MIC, area under the drug serum concentration–time curve over 24 h to the MIC

### Primary outcome

**Fig. 2:**
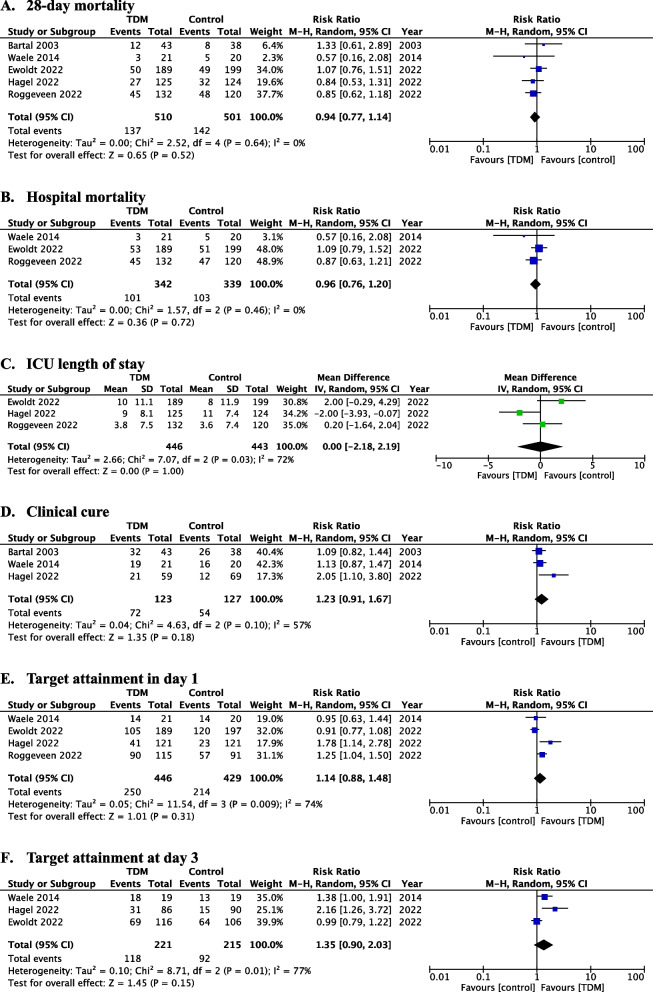
Forest plot of TDM and control group comparing 28-day mortality (**A**), hospital mortality (**B**), ICU length of stay (**C**), clinical cure (**D**), target attainment in 24 h (**E**), and target attainment at day 3 (**F**). *CI* confidence interval, *df* degrees of freedom, *M-H* Mantel–Haenszel test, *IV* inverse variance, *RR* risk ratio, *TDM* therapeutic drug monitoring

All five studies had 28-day mortality data available. Of the 1011 patients, 26.9% (137 of 510 patients) in the TDM group and 28.3% (142 of 501 patients) in the control group died within 28 days of randomization (Fig. 2, Table 2). The RR was 0.94 (95% CI, 0.77–1.14). Heterogeneity was not observed for the primary outcome (*I*^2^ = 0%, *χ*^2^ = 2.52, *P* = 0.64).

**Table 2:** Summary of finding table in included RCT studies

Outcomes	Certainty assessment					No. of patients		Effect		
	No. of studies	Risk of bias	Inconsistency	Indirectness	Imprecision	TDM	Placebo	Relative (95%CI)	Absolute (95%CI)	Certainty
28-Day mortality	5	Not serious	Not serious	Not serious	Serious^a^	137/510 (26.9%)	142/501 (28.3%)	RR 0.94 (0.77–1.14)	17 fewer per 1000 (from 65 fewer to 42 more	⨁⨁⨁◯ Moderate
Hospital mortality	3	Very serious^b^	Not serious	Not serious	Serious^a^	101/342 (29.5%)	103/339 (30.4%)	RR 0.96 (0.76 to 1.20)	12 fewer per 1000 (from 73 fewer to 61 more)	⨁◯◯◯ Very low
ICU length of stay	3	Not serious	Serious^c^	Not serious	Serious^d^	446	443		MD 0 (2.18 lower to 2.19 higher)	⨁⨁◯◯ Low
Clinical cure	3	Very serious^b^	Serious^c^	Serious^e^	Very serious^f^	72/123 (58.5%)	54/127 (42.5%)	RR 1.23 (0.91 to 1.67)	98 more per 1000 (from 38 fewer to 285 more)	⨁◯◯◯ Very low
Target attainment in day 1	4	Very serious^b^	Serious^c^	Serious^e^	Serious^g^	250/446 (56.1%)	214/429 (49.9%)	RR 1.14 (0.88 to 1.48)	70 more per 1000 (from 60 fewer to 239 more)	⨁◯◯◯ Very low
Target attainment at day 3	3	Very serious^b^	Serious^c^	Serious^e^	Very serious^f^	118/221 (53.4%)	92/215 (42.8%)	RR 1.35 (0.90 to 2.03)	150 more per 1000 (from 43 fewer to 441 more)	⨁◯◯◯ Very low

*CI* confidence interval, *MD* mean difference, *RR* risk ratio

^a^95% confidence interval for risk ratio includes 1.0

^b^High risk of the selection of the reported result

^c^The value of *I*^2^ is over 50% which should be considered as serious

^d^95% confidence interval for MD includes 0

^e^The definition of the outcome varies between studies

^f^95% confidence interval for risk ratio includes 1.0 which means no effect for outcome and 1.25 which means serious benefit, and the number of events is under the optimal information size assuming the relative risk reduction of 25%

^g^95% confidence interval for risk ratio includes 1.0 which means no effect for outcome and 1.25 which means serious benefit

### Secondary outcomes

In the meta-analysis of included RCTs, the pooled RRs in TDM group were 1.14 (95% CI, 0.88–1.48) for target attainment at day 1, 1.35 (95% CI, 0.90–2.03) for target attainment at day 3, 1.23 (95% CI, 0.91–1.67) for clinical cure, and 0.96 (95% CI: 0.76–1.20) for in-hospital mortality. Severe heterogeneity was observed in target attainment at day 1 (*I*^2^ = 74%, *χ*^2^ = 11.54, *P* = 0.009) and day 3 (I^2^ = 77%, χ^2^ = 8.71, *P* = 0.01) (Fig. 2, Table 2). In the meta-analysis of RCTs for length of stay in the ICU, mean difference in the TDM group was 0 day (95% CI, − 2.18–2.19), and severe heterogeneity was observed (*I*^2^ = 72%, *χ*^2^ = 7.07, *P* = 0.03). Regarding safety outcomes, Bartal’s study reported nephrotoxicity in 2 patients (4.7%) in the TDM group and 8 patients (21.1%) in the control group, while Hagel reported adverse events in 20 patients (15.7%) in the TDM group and 27 patients (21.3%) in the control group. However, no meta-analysis was performed since there were no safety outcome that could be integrated between these two studies.

### Subgroup analysis

Subgroup analysis included several analyzable studies on beta-lactam antibiotics and meta-analysis was performed. In two studies of 290 patients included for the analysis of 28-day mortality, there was no significant difference between two groups (TDM group: 20.5% [30 of 146 patients], control group: 25.7% [37 of 144 patients], RR: 0.94 [95% CI, 0.77–1.14]) (Additional file 1: Fig. S2). The pooled RRs in TDM group were 1.25 (95% CI, 0.91–1.73) for target attainment at day 1, 1.67 (95% CI, 0.98–2.85) for target attainment at day 3, and 1.47 (95% CI, 0.67–3.20) for clinical cure.

### Risk of bias assessment

**Fig. 3:**
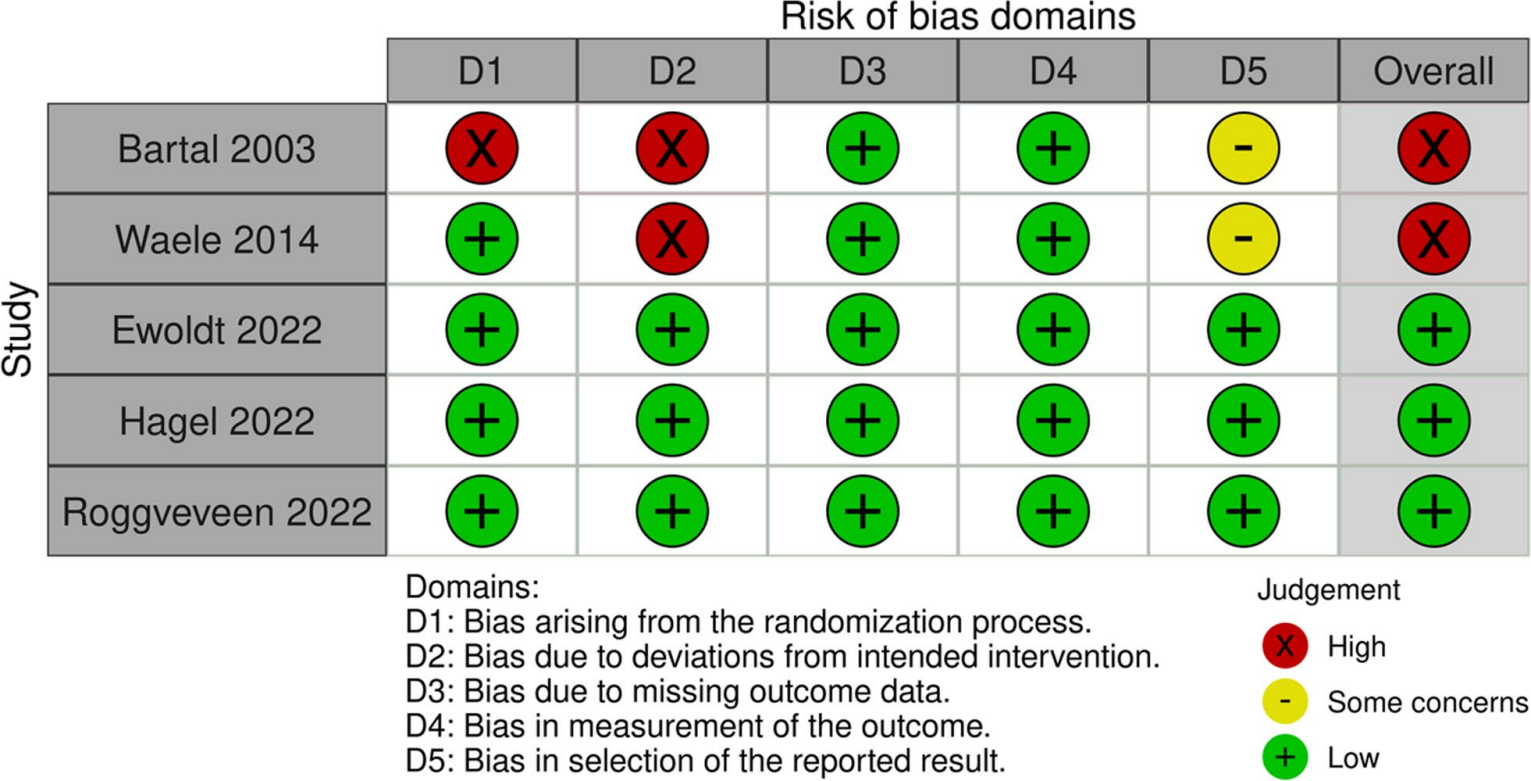
Summary of risk of bias for primary outcome in the included studies

For primary outcome, one RCT was rated as high risk owing to bias arising from the randomization process (Fig. 3). In the study by Bartal, patients were allocated to study groups by the last digit of their national identification card number, causing a possible bias at the point of allocation concealment. Furthermore, two RCTs by Bartal and Waele were rated as high risk owing to deviations from the intended intervention. This occurred because, in both studies, the participants or clinicians might have known what groups patients were assigned to. Furthermore, whether appropriate analyses, such as ITT analyses, were performed to consider allocation effects was unclear. Consequently, the quality of evidence for primary outcome was rated as moderate; the grade was lowered by one point owing to imprecision of the study (the CIs crossed clinical decision thresholds) (Table 2). The quality of the evidence for each secondary outcome was very low to low.

### Publication bias

The presence of publication bias was considered for the primary outcome of included RCT studies as indicated by the funnel plot (Additional file 1: Fig. S1). It was visually symmetric, indicating a low possibility of heterogeneity and reporting bias. However, the number of published studies was small [24].

## Discussion

This systematic review and meta-analysis showed no significant difference in 28-day mortality between TDM-based regimens and standard protocols for the administration of antibiotics in critically ill patients. Similarly, target attainment, clinical cure, in-hospital mortality, and length of stay in the ICU were not associated with TDM-based regimens.

Previous studies have performed systematic reviews and meta-analysis evaluating TDM-based regimens of antibiotics compared to standard protocols for infectious patients. However, these studies were restricted by limited antibiotic types, inclusion of cohort studies, or inclusion of non-critically ill patients such as febrile neutropenic patients [31, 32]. In particular, recent systematic review and meta-analysis, which investigated the clinical efficacy of beta-lactam antibiotics in critically ill patients, included 11 studies despite limiting the type of antibiotics, making the analysis broader than our study [18]. However, only four of them were RCTs, and the remaining seven were retrospective observational studies. Furthermore, two of these retrospective observational studies were conference abstracts. Two of the RCTs were included in our study as well, but the other two were studies since one was unclear whether they clearly fit the definition of critically ill patients, and another did not have applicable outcome. Two of the RCTs included in our study were published after the publication of this study, resulting in many of the included RCTs being different. To our knowledge, our systematic reviews and meta-analysis is the first to only include RCTs and critically ill patients in evaluating the efficacy of TDM in all antibiotics.

In a systematic review examining the efficacy of TDM-guided dosing regimens of beta-lactam antibiotics, the 28-day mortality was not significantly different, which is similar to the results of our study [18]. Factors that influence the clinical outcomes such as 28-day mortality include whether drug concentrations in the blood are within the appropriate range to target the organisms causing the infection [33, 34]. In these studies, target attainment was significantly higher in the TDM group; however, the target was set differently because one study used continuous infusion of meropenem. Furthermore, the number of days was not set as a criterion for analysis, and it might pose a potential risk of greater variability in the results compared to that with a strictly defined time point. To avoid these ambiguities, we set up target attainment in day 1 and day3 as the outcome in our study. However, there was no difference between the two groups with respect to target attainments, while it is not so surprising as to day 1 since day 1 is the point at which the first blood levels are measured, and dose adjustments are made based on those results. In the individual analysis of target attainment, Ewoldt's results for both day 1 and day 3 were inversely oriented toward the other studies. This study examined the effectiveness of the optimization by model-informed precision dosing using TDM, and this study design is slightly different from the usual antibiotic dosing strategy based on TDM. Therefore, these results may be one of the reasons for the lack of valid results in the meta-analysis. The study also used two antibiotic agents, beta-lactam antibiotics and ciprofloxacin, and integrated outcomes, but these may not have been significant in our study. As a result, clinical outcomes such as 28-day mortality and clinical cure may not have differed.

Furthermore, the impact of achieving target attainment on clinical outcomes has not yet been determined. A study investigating the achievement of beta-lactam antibiotic concentration targets using TDM for critically ill patients showed that optimal exposure of unbound beta-lactams is not achieved in a significant proportion of the patients. However, failure to achieve PK/PD targets was not associated with negative clinical outcomes, suggesting that there was a discrepancy between target attainment and clinical outcomes [35]. Conversely, the DALI study, which examined beta-lactam antibiotic dosing in critically ill patients and the association between antibiotic concentrations and clinical outcome, revealed that the effect of increasing *f*T > MIC was more significant in patients with lower Acute Physiology and Chronic Health Evaluation II severity score [12]. Therefore, comparisons between TDM-based regimens and standard protocols for groups with different severities should be conducted in future studies.

The targets set for blood concentrations varied among studies included in the meta-analysis. For quinolones, glycopeptides, and aminoglycosides, studies commonly used AUC/MIC for target attainment, whereas two different indices, MIC and 4 × MIC, were used for beta-lactams, depending on the study. These differences may be one of the sources of variation and heterogeneity among studies, although many target attainment breakpoints are considered to be set by convention or expert opinion [36]. A study investigating the difference in efficacy among critically ill patients treated with meropenem or piperacillin–tazobactam divided the target attainment into three groups: < 100% *f*T > MIC, 100% *f*T > MIC < 4 × MIC, and 100% *f*T > 4 × MIC. This study showed that 100% *f*T > MIC was associated with improved outcome, whereas no significant benefit was observed in the 100% *f*T > 4 × MIC group [37]. Although the target of the included studies was different as they included both MIC and 4 × MIC, the goal of TDM might not necessarily be to achieve 4 × MIC, even in critically ill patients. Furthermore, owing to the strict criteria of reaching 4 × MIC, the included studies also showed that only 33.9% of the TDM group met target attainment on day 1 in a study by Hagel, and this may have been the reason why the meta-analysis did not show differences in target attainment. In addition, other studies did not present separate results for beta-lactams; thus, their effects were unknown. Accordingly, future studies may have to focus on beta-lactams and set attainable targets or compare the clinical and pharmacological outcomes between the *f*T > MIC and *f*T > 4 × MIC targets in continuous and intermittent infusion setting. Furthermore, a previous systematic review and meta-analysis evaluating the association between AUC/MIC and clinical outcomes for vancomycin was inconsistent owing to the lack of standardized methods and insufficient data, thereby failing to obtain a positive outcome with AUC/MIC [38]. Even in our meta-analysis, no individual data were obtained for vancomycin and ciprofloxacin using AUC/MIC, and separate studies are required to investigate TDM-based regimen using AUC/MIC.

In summary, our meta-analysis did not find significant results for any of the outcomes, suggesting that this was due to inconsistent protocol and outcome settings across studies rather than a negative effect of TDM-based antibiotic regimen. In addition, there were several favorable results for TDM-based regimen, and no clear harms from TDM were observed. Therefore, our study does not reject TDM-based antibiotic strategies, and it is believed that effective results would be obtained if they are targeted to optimal clinical cases.

Our study had several limitations. First, because we limited our inclusion criteria to only RCT studies and critically ill patients, only five studies were included for the meta-analysis, which may have resulted in reduced statistical power. If we detect a significant effect from a 26.9% mortality rate in the TDM and 28.3% in the control groups, 16,146 samples would be required for each group (alpha error; 0.05, beta error; 0.80). However, variation in patient background and inclusion of non-RCTs may ultimately lead to heterogeneity of results and compromise the certainty of the obtained results. It is necessary to conduct RCTs on TDM that clearly target sepsis in the future. Second, some of our study outcomes showed significant statistical heterogeneity, which may be caused by the variation of definitions in each study. Particularly, Waele defined clinical cure as an improvement of clinical signs, whereas Bartel used a negative culture and normalization of white blood cell counts, and Hagel used no longer requiring additional antibiotics. In such cases, a narrower definition may narrow the patient population and cause difficulties in comparing the same population. If two studies with high bias risk are excluded, only the study by Hagel in which clinical cure is strictly defined is remained, and the RR would show significant difference (RR, 2.05; 95% CI, 1.10–3.80). Therefore, a clear definition of outcomes including clinical cure and target attainment must be adequately defined in future studies. Third, we used only a limited number of search engines for the literature search in our study. In particular, we did not use EMBASE, which has the potential to provide more extensive and systematic search results [added reference]. In the future, it is necessary to plan a search strategy that includes EMBASE in order to collect more appropriate literature.

## Conclusions

For critically ill patients, TDM-based antibiotic regimens were not associated with 28-day mortality, in-hospital mortality, clinical cure, target attainment at day 1 and day 3, and length of stay in the ICU when compared with standard protocols. Further RCTs in which outcomes are clearly defined, include disease severity, and focus on sepsis patients are warranted to verify the true efficacy of TDM.

### Supplementary Information

**Additional file 1: Table S1. **Literature search strategy for each database. **Figure S1.** Forest plot of TDM and control group in beta-lactam antibiotics. **Figure S2.** Funnel plot for the publication bias assessment.

## Data Availability

The datasets used and analyzed during the current study are available from the corresponding author upon reasonable request.

## Abbreviations

TDM: Therapeutic drug monitoring
ICU: Intensive care unit
PK/PD: Pharmacokinetics/pharmacodynamics
RCT: Randomized control trial
GRADE: Grading of Recommendations Assessment, Development, and Evaluation
RR: Risk ratios
CI: Confidence intervals
SD: Standard deviation
IQR: Interquartile
AUC: Area under the curve
MIC: Minimum inhibitory concentration
ITT: Intention to treat
