# Supplementary material for: Efficacy of therapeutic drug monitoring-based antibiotic regimen in critically ill patients: a systematic review and meta-analysis of randomized controlled trials
Source: J Intensive Care. 2023 Nov 8;11:48. doi: 10.1186/s40560-023-00699-8 (PMC10631080; doi:10.1186/s40560-023-00699-8)
Supplement: Supplementary file 1 — Additional file 1: Table S1. Literature search strategy for each database. Figure S1. Forest plot of TDM and control group in beta-lactam antibiotics. Figure S2. Funnel plot for the publication bias assessment. [file 40560_2023_699_MOESM1_ESM.pdf]

Table S1. Literature search strategy for each database

Key words

Sepsis, Systemic Inflammatory Response Syndrome, Multiple Organ Failure, Critically ill, Therapeutic Drug Monitoring, Randomized controlled trial

PubMed search strategy（Search date; 17 November 2022）

	Search formula	Hit No
#1	Sepsis[mesh] or sepsis[tiab] or “Systemic Inflammatory Response Syndrome”[mesh] or “Systemic Inflammatory Response Syndrome”[tiab] or SIRS[tiab] or “septic shock”[tiab] or “Multiple Organ Failure”[mesh] or “Multiple Organ Failure”[tiab] or MOF[tiab] or “Critical Illness”[mesh] or “Critically ill”[tiab]	304,499
#2	“Drug Monitoring”[mesh] or “Drug monitoring”[tiab] or “TDM”[tiab]	32,312
#3	Randomized controlled trial[pt] or controlled clinical trial[pt] or randomized[tiab] or placebo[tiab] or drug therapy[sh] or randomly[tiab] or trial[tiab] or groups[tiab] not (animals [mh] not humans [mh])	4,876,469
#4	#1 and #2 and #3	719

CENTRAL search strategy（Search date; 17 November 2022）

	Search formula	Hit No
#1	MeSH descriptor: [Sepsis] explode all trees	5,002
#2	(sepsis):ti,ab,kw	12,608
#3	MeSH descriptor: [Systemic Inflammatory Response Syndrome] explode all trees	5,404
#4	(systemic inflammatory response syndrome):ti,ab,kw	1,496
#5	MeSH descriptor: [Shock, Septic] explode all trees	1,092
#6	(shock, septic):ti,ab,kw	3,635
#7	MeSH descriptor: [Multiple Organ Failure] explode all trees	442
#8	(Multiple Organ Failure):ti,ab,kw	2,059
#9	MeSH descriptor: [Critical Illness] explode all trees	2,682
#10	("critically ill"):ti,ab,kw	7,976
#11	#1 or #2 or #3 or #4 or #5 or #6 or #7 or #8 or #9 or #10	25,293
#12	MeSH descriptor: [Drug Monitoring] explode all trees	1,918
#13	("drug monitoring"):ti,ab,kw	3,379
#14	(TDM):ti,ab,kw	409
#15	#12 or #13 or #14	3,575
#16	#11 and #15	138

Igaku-Chuo-Zasshi search strategy（Search date; 17 November 2022）

	Search formula	Hit No
#1	(敗血症/TH or 敗血症/TA) and (PT=会議録除く)	26,408
#2	(ショック-敗血症性/TH or 敗血症性ショック/TA) and (PT=会議録除く)	7,280
#3	(全身性炎症反応症候群/TH or 全身性炎症反応症候群/TA)	21,500

	and (PT=会議録除く)	
#4	((多臓器不全/TH or 多臓器不全/TA)) and (PT=会議録除く)	6,871
#5	#1 or #2 or #3 or #4	33,843
#6	(ドラッグモニタリング/TH or ドラッグモニタリング/TA or TDM/TA or 薬物血中濃度/TA or 薬物モニタリング/TA or ((薬物/TH or Drug/AL) and Monitoring/TA)) and (PT=会議録除く)	3,111
#7	ランダム化比較試験/TH or 準ランダム化比較試験/TH or ランダム化/AL or 無作為化/AL or 比較試験/AL or 臨床試験/AL or プラセボ/AL or 対照/AL or コントロール/AL or 臨床研究/AL or 治験/AL	395,358
#8	#5 and #6 and #7	6

Figure S1. Forest plot of TDM and control group in beta-lactam antibiotics.

(A) 28-day mortality

Study or Subgroup	TDM Events	TDM Total	Control Events	Control Total	Weight	Risk Ratio M-H, Random, 95% CI
Waele 2014	3	21	5	20	10.7%	0.57 [0.16, 2.08]
Hagel 2022	27	125	32	124	89.3%	0.84 [0.53, 1.31]
**Total (95% CI)**		**146**		**144**	**100.0%**	**0.80 [0.53, 1.23]**
Total events	30		37			

Heterogeneity: $Tau^2 = 0.00$; $Chi^2 = 0.30$, df = 1 (P = 0.58); $I^2 = 0\%$
Test for overall effect: Z = 1.01 (P = 0.31)

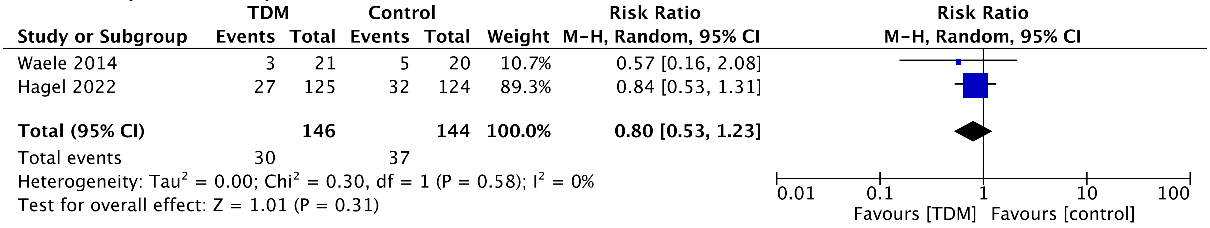

(B) Target attainment in 24 hours

Study or Subgroup	TDM Events	TDM Total	Control Events	Control Total	Weight	Risk Ratio M-H, Random, 95% CI
Waele 2014	14	21	14	20	28.7%	0.95 [0.63, 1.44]
Roggeveen 2022	49	58	33	47	44.3%	1.20 [0.97, 1.49]
Hagel 2022	41	121	23	121	27.0%	1.78 [1.14, 2.78]
**Total (95% CI)**		**200**		**188**	**100.0%**	**1.25 [0.91, 1.73]**
Total events	104		70			

Heterogeneity: $Tau^2 = 0.05$; $Chi^2 = 5.04$, df = 2 (P = 0.08); $I^2 = 60\%$
Test for overall effect: Z = 1.36 (P = 0.17)

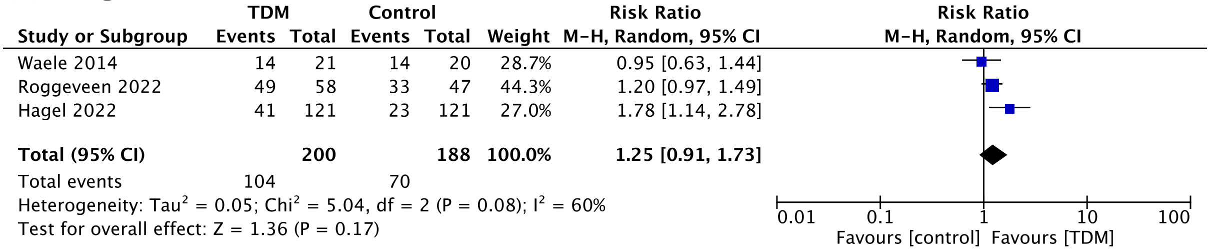

(C) Target attainment at day 3

Study or Subgroup	TDM Events	TDM Total	Control Events	Control Total	Weight	Risk Ratio M-H, Random, 95% CI
Waele 2014	18	19	13	19	58.0%	1.38 [1.00, 1.91]
Hagel 2022	31	86	15	90	42.0%	2.16 [1.26, 3.72]
**Total (95% CI)**		**105**		**109**	**100.0%**	**1.67 [0.98, 2.85]**
Total events	49		28			

Heterogeneity: $Tau^2 = 0.10$; $Chi^2 = 2.95$, df = 1 (P = 0.09); $I^2 = 66\%$
Test for overall effect: Z = 1.88 (P = 0.06)

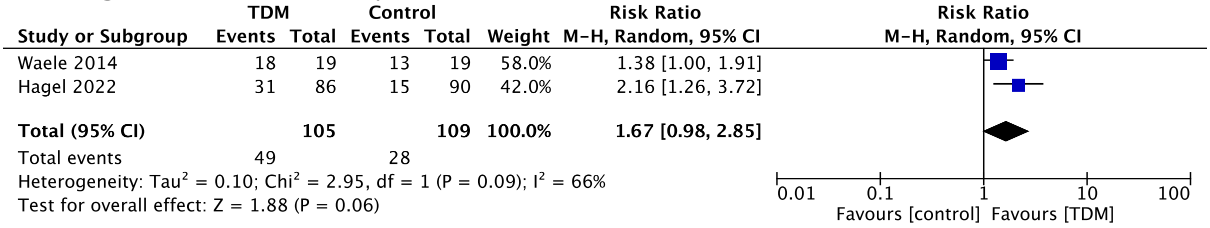

(D) Clinical cure

Study or Subgroup	TDM Events	TDM Total	Control Events	Control Total	Weight	Risk Ratio M-H, Random, 95% CI
Waele 2014	19	21	16	20	56.3%	1.13 [0.87, 1.47]
Hagel 2022	21	59	12	69	43.7%	2.05 [1.10, 3.80]
**Total (95% CI)**		**80**		**89**	**100.0%**	**1.47 [0.67, 3.20]**
Total events	40		28			

Heterogeneity: $Tau^2 = 0.26$; $Chi^2 = 5.52$, df = 1 (P = 0.02); $I^2 = 82\%$
Test for overall effect: Z = 0.96 (P = 0.34)

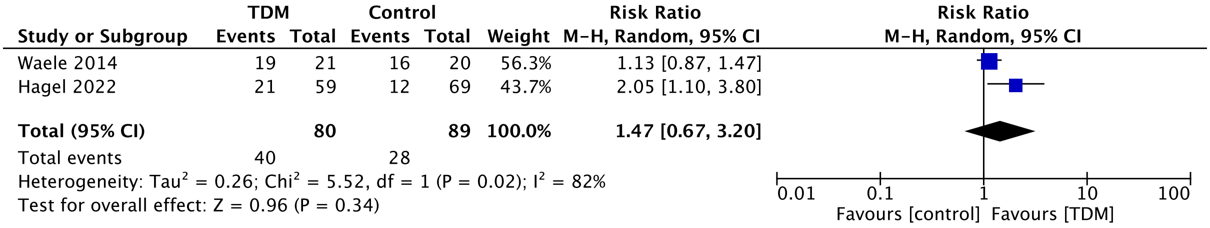

CI, confidence interval; df, degrees of freedom; M-H, Mantel-Haenszel Test; RR, risk ratio; TDM, therapeutic drug monitoring.

Figure S2. Funnel plot for the publication bias assessment

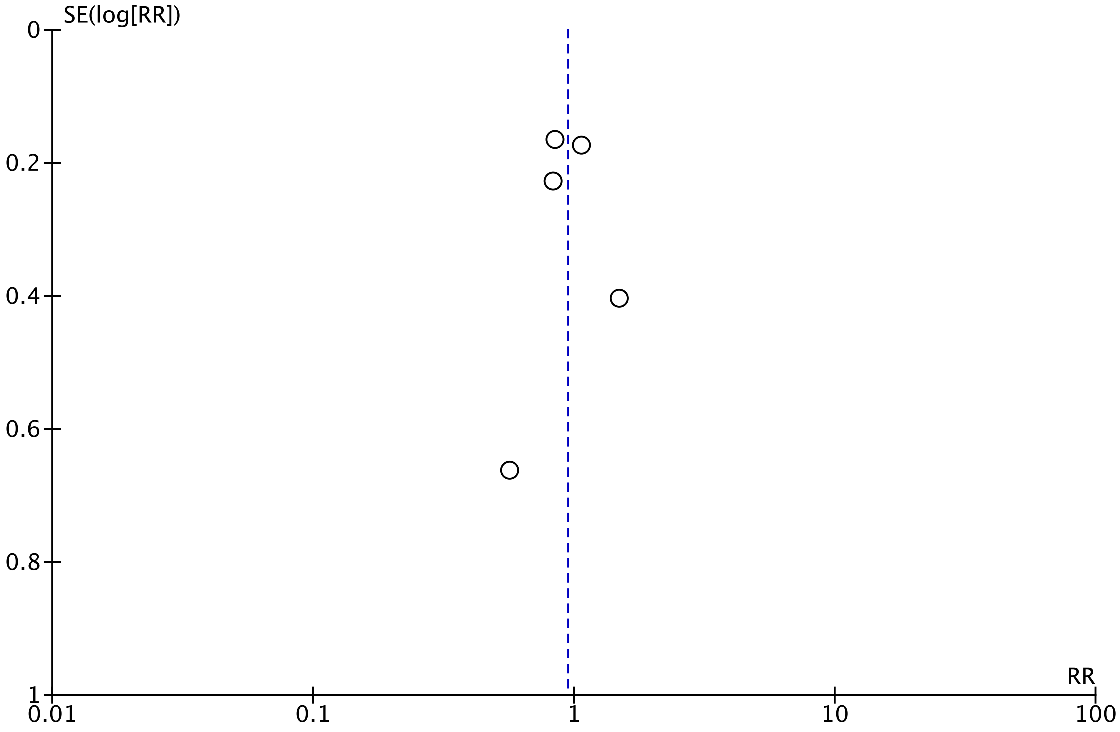

RR, risk ratio; SE, standard error
